# Association between number of previous malaria episodes and treatment failure among children and adults with laboratory-confirmed Plasmodium infection treated with pyronaridine-artesunate in Gabon: a retrospective cohort study

**DOI:** 10.1186/s12936-026-06147-w

**Published:** 2026-09-17

**Authors:** Ghyslain Mombo-Ngoma, Augusto Meneguim, Rella Zoleko Manego, Francine Ntoumi, Diane Egger-Adam, Jangsik Shin, Ayola Akim Adegnika, Sarah Arbe-Barnes, Isabelle Borghini-Fuhrer, Stephan Duparc, Peter G. Kremsner, Michael Ramharter, Jackie Cook, Mirjam Groger

**Affiliations:** 1 Centre de Recherches Médicales de Lambaréné (CERMEL), Lambaréné, Gabon; 2 Drug Implementation Research Group, Bernhard Nocht Institute for Tropical Medicine, and I. Department of Medicine, University Medical Centre Hamburg-Eppendorf, Hamburg, Germany; 3 Center for Tropical Medicine, Bernhard Nocht Institute for Tropical Medicine & I Dept. of Medicine University Medical Center Hamburg-Eppendorf, Hamburg, Germany; 4 Fondation Congolaise pour la Recherche Médicale (FCRM), WHO-AFRO Campus Djoué, Hospital of Talangai (North) of Brazzaville, Brazzaville, Republic of Congo; 5 Institut für Tropenmedizin, University of Tübingen, Tübingen, Germany; 6 Shin-Poong Pharmaceutical Co. Ltd, Seoul, Korea; 7 German Center for Infection Research (DZIF), Partner Site Tübingen, Tübingen, Germany; 8 Artemida Pharma, Stevenage, UK; 9 Medicines for Malaria Venture, Geneva, Switzerland; 10 German Center for Infection Research (DZIF), Partner Site Hamburg–Lübeck–Borstel–Riems, Hamburg, Germany; 11 London School of Hygiene and Tropical Medicine, London, UK

**Keywords:** Malaria, Plasmodium infection, Pyronaridine-artesunate, Recrudescence, Recurrence, Treatment failure, Prior infection

## Abstract

**Background:**

Malaria remains a public health challenge in Gabon. Pyronaridine-artesunate is an effective oral treatment for uncomplicated malaria. In real-world context, treatment failures, though still rare, are expected to be more frequent than in clinical trials. Prior malaria episodes have been suggested as risk factor for treatment failure. However, most evidence relies on self-reported history, which is prone to bias.

**Methods:**

A secondary data analysis of the CANTAM study, a prospective multicenter single arm open-label phase IIIB/IV study assessing the safety and efficacy of pyronaridine–artesunate in real-world context, was conducted. Adults and children weighing ≥ 5 kg with uncomplicated malaria were included. Data from the Centre de Recherches Médicales de Lambaréné (CERMEL), Gabon, were used in this sub-group analyses to assess the impact of previous malaria episodes on the risk for treatment failure, measured both as recurrence and recrudescence. Multivariate logistic regression models were used to adjust for potential confounders.

**Results:**

From June 2017 to March 2019, 2,082 patients were enrolled in Gabon; 1,969 were included in the intention-to-treat and 1,687 in the per-protocol analysis. Adults comprised 22.6% and males 53.7% of participants. In the per-protocol population, 129 (7.7%) participants experienced recurrence from which 22 (1.3%) were recrudescence. Repeated malaria episodes accounted for 294 (17.4%) of the malaria cases. Each one-year increase in age was associated with 11% decrease in the odds of recurrence (OR 0.89, 95%CI 0.86–0.92; p-value < 0.001) and recrudescence (OR 0.89, 95%CI 0.81–0.97; p-value 0.007). Previous malaria was associated with recurrence (OR 1.96; 95%CI 1.31–2.94; p-value 0.001) and recrudescence (OR 2.44; 95%CI 1.01–5.94; p-value 0.049) in the per-protocol multivariate analysis. The association between prior malaria and treatment failure was modified by age, indicating that treatment failure was less associated with previous exposure in older children.

**Conclusions:**

Previous malaria episodes may constitute a surrogate marker for increased risk of recurrence and recrudescence, particularly in younger children. This association may reflect increased vulnerability or transient alterations in host immunity, although other unmeasured factors may also contribute. This understanding could improve malaria management in high-transmission settings, highlighting the need for targeted follow-up of patients at highest risk of treatment failure.

## Introduction

According to the World Health Organization (WHO), there were around 282 million malaria cases and 610,000 deaths globally in 2024[1]. Among those, 94% were reported in Africa, which also harbored 95% of global malaria deaths. Gabon, with a population of 2,484,788 in 2024, presented around 475,500 malaria cases, with an incidence rate of 187 cases per 1,000 population at risk [1].

Uncomplicated malaria should be treated without delay to prevent progression to severe disease which can lead to organ dysfunction and mortality [2]. The current standard of care for uncomplicated malaria is artemisinin-based combination therapies (ACT)s. In a more recently registered ACT, artesunate was partnered with pyronaridine in a fixed-dose combination three-day oral therapy for use in infants, children and adults to treat acute, uncomplicated malaria. This fixed-dose combination was studied in both healthy volunteers and patients with uncomplicated malaria [3]. Considering the per-protocol polymerase chain reaction (PCR)-corrected adequate clinical and parasitological response (ACPR) on day 28 for uncomplicated *Plasmodium falciparum* malaria, an open-label sequential dose-escalation study showed an efficacy of 100% among children at all dose levels tested [4]. A subsequent randomized non-inferiority trial demonstrated an efficacy of 99.5% in children and adults [5], and an open-label randomized controlled trial evidenced an efficacy of 97.4% in children [6]. Furthermore, a multicenter, single-arm, open-label, cohort-event monitoring Phase IIIb/IV study was conducted in four Central African countries and in Côte d’Ivoire. The protocol replicated real-world clinical practice as closely as possible and assessed the real-world effectiveness of pyronaridine-artesunate with repeated dosing where appropriate [7]. While in this study the PCR-corrected cure rate in the per-protocol population was 98.6% on day 28, it was 90.9% in the intention-to-treat population, reflecting real-world effectiveness rather than efficacy [7]. Additionally, a post-hoc analysis suggested that the combination presented an effectiveness of at least 96.3% against mono-infections with *P. malariae*, *P. ovale* spp., and mixed-*Plasmodium* infections in real-world settings [8]. The weight-based dosage regimen in granule labeling for pediatric patients is also strongly supported by pharmacokinetic analyses [9]. Finally, recent evidence supports the use of pyronaridine-artesunate in pregnant women in their second and third trimesters [10].

Treatment failure can be caused by a variety of factors, including those related to the parasite and the host. Factors potentially associated with treatment failure include younger age, especially < 5 years [11–13], female sex [14], lack of health insurance [15], rural residence [16], severe anemia [13, 17], vomiting after treatment [11], drug formulation [7], high parasitemia [11], delayed treatment [18], non-adherence to treatment [19], and drug resistance [20]. Although artemisinin partial resistance has been reported in other African countries, there is currently no evidence of its presence in Gabon [21–23]. Interestingly, having had previous malaria treatment has also been associated with treatment failure as evidenced by two prospective studies conducted in Thailand and Ethiopia [11, 24]. However, the studies assessing the role of previous malaria treatment as risk factor relied on self-report and is therefore prone to recall bias and misclassification. A review of the literature revealed no studies assessing documented prior treatment for laboratory-confirmed infections as a potential risk factor for treatment failure.

Therefore, a secondary data analysis was conducted using data from Gabon from the above-mentioned Phase IIIb/IV cohort-event monitoring study evaluating the real-world effectiveness of pyronaridine-artesunate [7]. This analysis assessed whether documented previous malaria treatment within the study period was associated with pyronaridine–artesunate treatment failure among children and adults with laboratory-confirmed *P. falciparum* infection in a real-world setting.

## Methods

### Study design

A secondary analysis was conducted using the Gabon data from the prospective multicentric, single-arm, open-label *Phase IIIB/IV cohort event monitoring study to evaluate, in real life setting, the safety and tolerability in malaria patients of the fixed-dose artemisinin-based combination therapy Pyramax® (pyronaridine-artesunate) (NCT03201770).* More details about the original study can be found in previous reports [7, 8, 25].

### Participants

The study population included adults and children of either sex, weighing at least 5 kg, attending the trial site with RDT- or microscopy-confirmed acute uncomplicated malaria as per local standard of care and able to take oral medication. Exclusion criteria comprised clinical signs or symptoms of hepatic injury or known severe liver disease, known allergy to artemisinin and/or to pyronaridine, known pregnancy, lactation, severe malaria as defined by WHO [2], use of pyronaridine-artesunate in the previous 28 days, and patients that the investigator considered to be at particular risk if participating in the study. To reflect real-world practice, patients could be included more than once in the study, following a 28-day washout period between consecutive pyronaridine–artesunate treatments. The participants had not been vaccinated.

### Study procedures

The study comprised visits on days 0, 7 (± 1), and 28 (± 2). The total follow-up period for a single participant episode was 28 (± 2) days. On day 0, every participant visiting the study health facility for whom a diagnosis of uncomplicated malaria (according to WHO Criteria [26]) was suspected, underwent standard-of-care diagnostic (rapid diagnostic test or microscopy). If the presence of malaria and other eligibility criteria were confirmed, patients were enrolled. The first dose of fixed-dose pyronaridine-artesunate (Shin-Poong Pharmaceutical, Seoul, South Korea) was administered under Direct Observational Treatment conditions according to body weight. Thereafter, participants were instructed to take their second and third doses orally every 24 h from the first administration under real-world conditions (including unsupervised medication intake at home and without regard to food intake). For children, caretakers were instructed on how to administer the daily dose. All participants received bed nets. On day 7 (± 1 day), a community health worker (CHW) conducted a home visit where they assessed the current clinical condition and treatment. On day 28 (± 2 days), the CHW conducted a second home visit, which constituted the final study endpoint assessment of treatment effectiveness. On this occasion, they also collected blood for thick blood smear and PCR genotyping, which were analyzed retrospectively only for those with recurrence on day 28. A patient was considered lost to follow-up if he could not be visited by the CHW before day 10. However, all reasonable efforts were made by the study site personnel to contact the subject for the day 28 visit, to determine the endpoint status and the reason for discontinuation/withdrawal. More details about the study procedures can be found in the original publication [7].

### Outcomes and study populations

The main outcome of this sub-analysis was treatment failure. This term covers recurrence, defined as a positive thick blood smear at or before day 28 (+ 2), and recrudescence, defined as reappearance of the same parasite genotype as assessed by PCR when comparing blood spot samples taken at baseline versus the time point of recurrence, using published criteria [27]. Recurrence and recrudescence were assessed in the intention-to-treat and per-protocol populations. The intention-to-treat population included all malaria episodes treated with at least 1 dose of study medication with confirmed positive parasitemia at baseline. The per-protocol population included all malaria episodes in the intention-to-treat population in patients who were treated with a full course of study medication, based on patient self-assessment of adherence, and had a day 28 effectiveness endpoint. Participants who vomited after study drug administration were excluded, except where vomiting occurred after the first dose and dosing was successfully repeated. Participants who received prior or concomitant medication with antimalarial activity that could interfere with the treatment outcome were also excluded. The trial populations were prespecified in the study protocol and statistical analysis plan of the original study [7]. Having a previous malaria episode within the study period was considered as the exposure. Participants could be included more than once in the study, as it evaluated malaria episodes, not individuals. A washout period of at least 28 days was maintained between two consecutive treatment cycles with pyronaridine-artesunate.

### Statistical analysis

Characteristics of the study population were summarized using descriptive statistics. Continuous variables were presented as median and interquartile range (IQR), and categorical variables as frequencies and proportions. Age, body mass index (BMI) and seasonality were considered as a priori confounders [28–31]. Age was categorized as < 5 or ≥ 5 years, in line with the standard epidemiological threshold reflecting higher vulnerability to malaria outcomes. Low BMI was defined based on the BMI percentiles per age group [32]. Peak season was defined as enrollments from October to March, the months associated with higher rainfall in Gabon. Associations between exposure, outcome, and categorical covariates were assessed using cross-tabulations and chi-square tests, and summarized using odds ratios (OR), 95% confidence intervals (95% CI), and p-values. Crude association between previous malaria episode (analyzed as both binary and three-category variables) and treatment failure (recurrence in the crude analysis and recrudescence in the PCR-corrected analysis) was assessed using logistic regression and summarized using odds ratios, 95% confidence intervals, and p-values. For the multi-category exposure, a test for trend of odds was performed to assess dose–response, and the Likelihood Ratio test was used to evaluate overall association. Age was modelled as a continuous variable in logistic regression analyses, as a linear trend with the outcome was observed. Effect modification was assessed by including interaction terms in logistic regression models and evaluating them using likelihood ratio tests comparing models with and without interaction terms. Significant interaction terms were retained in the final multivariate models. Multivariate analysis was conducted using logistic regression. Given the limited number of recrudescence events in the per-protocol population, adjustment was restricted to the three a priori specified potential confounders age, BMI, and seasonality. A mixed-effects logistic regression model with a random intercept for participant was explored to account for repeated observations. For the determination of treatment failure, participants with a missing parasitological assessment at day 28 were considered failures in the intention-to-treat population and excluded from the per-protocol population. Participants with missing PCR results were considered as recrudescence in the intention-to-treat population and excluded from the per-protocol population.

STATA v.17 was used for statistical analyses. The estimated post-hoc statistical power of this secondary analysis was 84% to detect an association, if present, assuming that participants receiving their first malaria treatment with pyronaridine-artesunate in the study had a 9% risk of treatment failure compared to 15% in participants with a previous episode [7, 11].

## Results

### Study participants

**Fig. 1:**
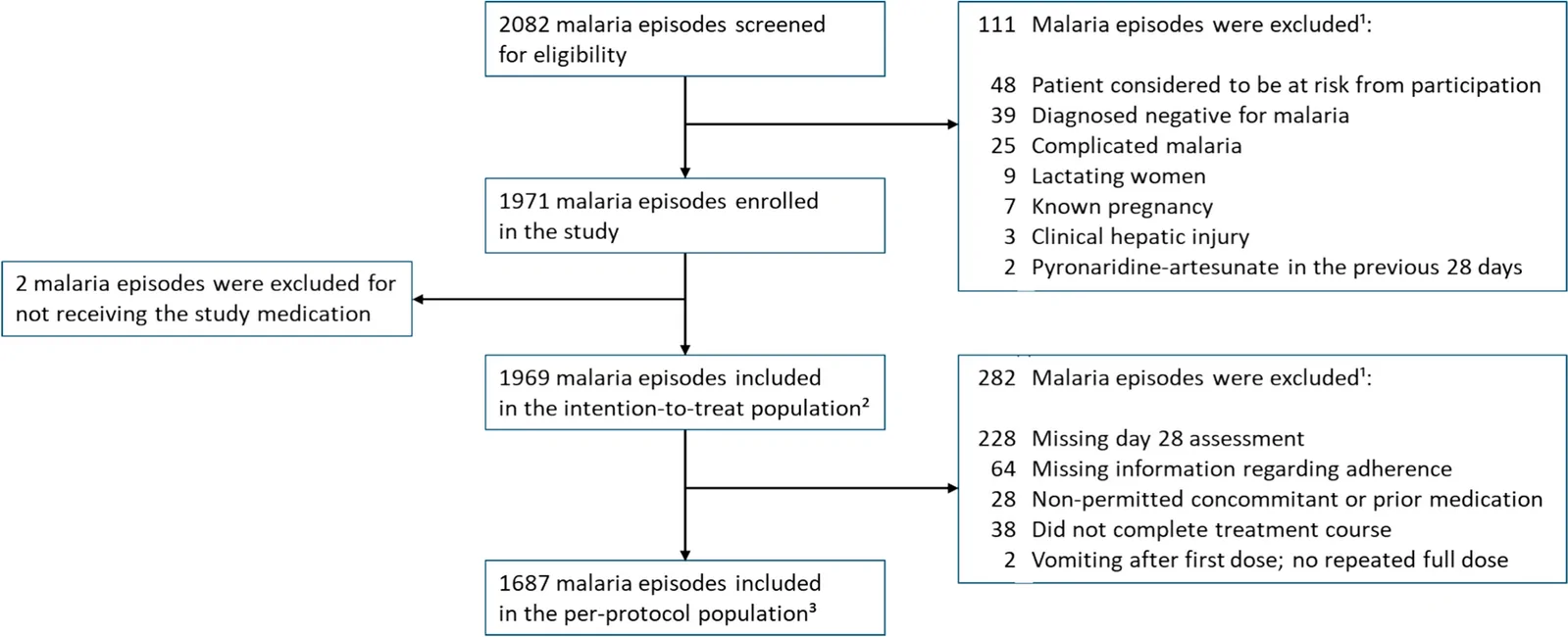
CONSORT diagram. ^1^ Multiple criteria may apply. ^2^ The intention-to-treat population included all malaria episodes treated with at least 1 dose of study medication with confirmed positive parasitemia at baseline. ^3^ The per-protocol population included all malaria episodes in the intention-to-treat population in patients who were treated with a full course of study medication (adherence per patient self-assessment), had a day 28 effectiveness endpoint, did not vomit after study drug administration (except where vomiting occurred after the first dose and dosing was successfully repeated), and who had no prior or concomitant medication, which had an antimalarial effect and so could interfere with the treatment outcome

The participant flow is described in Fig. 1. In Gabon, from 22 June 2017 to 11 March 2019, 2082 patients provided written informed consent for the study and underwent screening, as shown in Fig. 1. Overall, 1971 patients met the eligibility criteria and were enrolled in the study. Out of those, 2 did not receive the study medication. In this secondary analysis, 1969 participants were included in the intention-to-treat and 1687 in the per-protocol populations. In total, 1590 participants completed the 28-day follow-up period with all study visits. Although 379 participants discontinued the study as per protocol definition, a day 28 (± 2 days) blood sample for thick blood smear and PCR genotyping was collected from 223 of them.

### Descriptive analysis

The characteristics of participants are summarized in Table 1. In the intention-to-treat population, 375 (19.0%) participants presented recurrence from which 277 (14.1%) were recrudescence. In this population, missing blood smears/PCR were considered as treatment failures. Previous malaria episode during the study period was documented in 342 (17.4%) participants. The maximum number of episodes was nine which appeared in one participant. *Plasmodium falciparum* mono-infection predominated (98.4%), followed by co-infection with *P. ovale* spp. (1.0%), co-infection with *P. malariae* (0.5%), and triple co-infection (0.1%). Children < 5 years old accounted for 445 (22.6%), and male sex for 961 (53.7%) of participants. Human immunodeficiency virus (HIV) infection was reported by 4 (0.2%) participants, although 1899 (96.4%) did not know their status. A missed treatment dose was reported by only 38 (1.9%) participants. In the per-protocol population, 129 (7.7%) participants presented recurrence from which 22 (1.3%) were recrudescence, and 294 (17.4%) experienced a previous malaria episode during the study period.

**Table 1:** Characteristics of participants

Co-variable	Category	Intention-to-treat n (%) (N = 1,969)	Per-protocol n (%) (N = 1,687)
Treatment failure—recurrence	No	1,594 (81.0)	1,558 (92.4)
	Yes	375 (19.0)	129 (7.7)
Treatment failure—recrudescence	No	1,692 (85.9)	1,655 (98.7)
	Yes	277 (14.1)	22 (1.3)
	Missing	–	10 (0.6)
Previous malaria within cohort follow-up time	No	1,627 (82.6)	1,393 (82.6)
	Yes	342 (17.4)	294 (17.4)
Malaria episode within cohort follow-up time	1 st episode	1,623 (82.4)	1,386 (82.2)
	2nd episode	267 (13.6)	228 (13.5)
	3rd episode	46 (2.3)	45 (2.7)
	4th episode	19 (1.0)	16 (1.0)
	5th episode	8 (0.4)	6 (0.4)
	6th episode	2 (0.1)	2 (0.1)
	7th episode	2 (0.1)	2 (0.1)
	8th episode	1 (0.05)	1 (0.06)
	9th episode	1 (0.05)	1 (0.06)
Age (years)	Median (IQR)	10 (5–17)	10 (5–16)
	≥ 18	462 (23.5)	388 (23.0)
	5 to < 18	1,062 (53.9)	926 (54.9)
	< 5	445 (22.6)	373 (22.1)
Sex	Male	1,054 (53.5)	909 (53.9)
	Female	915 (46.5)	778 (46.1)
BMI	Not low	1,592 (80.9)	1,439 (85.3)
	Low	377 (19.2)	248 (14.7)
Treatment formulation	Tablet	1,332 (67.7)	1,147 (68.0)
	Granule	637 (32.4)	540 (32.0)
HIV	No	66 (3.4)	59 (3.5)
	Yes	4 (0.2)	4 (0.2)
	Unknown	1,899 (96.4)	1,624 (96.3)
Any missed dose reported by patient	No	1,867 (94.8)	1,687 (100.0)
	Yes	38 (1.9)	NA
	Missing	64 (3.3)	NA
Peak season*	No	720 (36.6)	635 (37.6)
	Yes	1,249 (63.4)	1,052 (62.4)

*Peak season was defined as episodes enrolled from October to March, the months associated with higher rainfall in Gabon. IQR, interquartile range; NA, not applicable

### Association between co-variables, treatment failure and previous malaria episodes

The association between each co-variable and treatment failure is described in Table 2. Younger age categories were progressively associated with higher odds of recurrence and recrudescence, as evidenced by linear trend. In the ITT analysis, younger age (< 5 years old) showed strong evidence of association with recurrence (OR: 2.30; 95%CI 1.66–3.18; p-value: < 0.001), while the evidence was weak for recrudescence (OR: 1.38; 95%CI 0.97–1.96; p-value: 0.077). In the per-protocol analysis, age < 5 years was associated with strong evidence to both recurrence (OR: 11.70; 95%CI 5.29–25.87; p-value: < 0.001) and recrudescence (OR: 7.61; 95%CI 1.72–33.72; p-value: 0.008). Considering age as continuous variable, each one-year increase in age was associated with 11% decrease in the odds of recurrence (OR: 0.89, 95%CI 0.86–0.92; p-value: < 0.001) and recrudescence (OR: 0.89, 95%CI 0.81–0.97; p-value: 0.007) in the per-protocol analyses. There was no evidence of association between treatment failure and sex, BMI, or seasonality.

**Table 2:** Distribution of participants per co-variable categories and association with recurrence, recrudescence, and previous malaria episode

Co-Variable	Category	Intention-to-treat			Per-protocol		
		Recurrence n (%) (N = 375)	OR (95%CI)	p-value	Recurrence n (%) (N = 129)	OR* (95%CI)	p-value
Age (years)	≥ 18	71 (15.4)	–	0.652	7 (1.8)	–	0.002
	5 to < 18	173 (16.3)	1.07 (0.79–1.45)		56 (6.1)	3.50 (1.58–7.78)	
	< 5	131 (29.4)	2.30 (1.66–3.18)	< 0.001	66 (17.7)	11.7 (5.29–25.87)	< 0.001
	Trend of odds^1^	–	1.57 (1.33 − 1.85)	< 0.001	–	3.06 (2.34–4.0)	< 0.001
Sex	Male	201 (19.1)	–	0.976	70 (7.7)	–	0.928
	Female	174 (19.0)	1.00 (0.79–1.26)		59 (7.6)	0.98 (0.67–1.43)	
BMI	Not low	302 (19.0)	–	0.861	102 (7.1)	–	0.038
	Low	73 (19.4)	1.03 (0.76–1.37)		27 (10.9)	1.60 (0.98–2.53)	
Peak season^2^	No	130 (18.1)	–	0.396	55 (8.7)	–	0.223
	Yes	245 (19.6)	1.11 (0.87–1.41)		74 (7.0)	0.80 (0.55–1.17)	

		Recrudescence n (%) (N = 277)	OR (95% CI)	p-value	Recrudescence n (%) (N = 22)	OR* (95%CI)	p-value
Age (years)	≥18	66 (14.3)	–	0.230	2 (0.5)	–	0.773
	5 to < 18	128 (12.1)	0.82 (0.60–1.13)		6 (0.7)	1.27 (0.25–6.30)	
	< 5	83 (18.7)	1.38 (0.97–1.96)	0.077	14 (3.79)	7.61 (1.72–33.72)	0.008
	Trend of odds^1^	–	1.19 (0.99–1.44)	0.064	–	3.49 (1.87–6.52)	< 0.001
Sex	Male	147 (14.0)	–	0.868	9 (1.0)	–	0.223
	Female	130 (14.2)	1.02 (0.79–1.33)		13 (1.7)	1.69 (0.67–4.51)	
BMI	Not low	226 (14.2)	–	0.737	19 (1.3)	–	0.903
	Low	51 (13.5)	0.95 (0.67–1.32)		3 (1.2)	0.93 (0.17–3.18)	
Peak season^2^	No	91 (12.6)	–	0.166	11 (1.8)	–	0.226
	Yes	186 (14.9)	1.21 (0.92–1.60)		11 (1.1)	0.60 (0.23–1.53)	

		Previous malaria n (%) (N = 342)	OR (95%CI)	p-value	Previous malaria n (%) (N = 294)	OR* (95%CI)	p-value
Age (years)	≥ 18	46 (10.0)	–	< 0.001	40 (10.3)	–	0.001
	5 to < 18	190 (17.9)	1.98 (1.40–2.78)		166 (17.9)	1.90 (1.32–2.75)	
	< 5	106 (23.8)	2.83 (1.94–4.11)	< 0.001	88 (23.6)	2.69 (1.79–4.03)	< 0.001
	Trend of odds^1^	–	1.62 (1.37–1.93)	< 0.001	–	1.59 (1.32–1.91)	< 0.001
Sex	Male	188 (17.8)	–	0.557	161 (17.7)	–	0.739
	Female	154 (16.8)	0.93 (0.73–1.19)		133 (17.1)	0.96 (0.74–1.24)	
BMI	Not low	254 (16.0)	–	0.001	240 (16.7)	–	0.051
	Low	88 (23.3)	1.60 (1.20–2.12)		54 (21.8)	1.39 (0.98–1.95)	
Peak season^2^	No	103 (14.3)	–	0.006	93 (14.7)	–	0.019
	Yes	239 (19.1)	1.42 (1.10–1.84)		201 (19.1)	1.38 (1.05–1.82)	

Percentages are calculated within each category. Odds ratios and p-values calculated via cross-tabulations and chi-square tests for binary variables and logistic regression for stratum-specific results for variables with > 2 categories

^1^Test for trend of odds considering 3 age categories; ^2^ Peak season was defined as episodes enrolled from October to March, the months associated with higher rainfall in Gabon

Additionally, younger age categories were also progressively associated with higher odds of multiple malaria episodes, as also evidenced by linear trend. Children aged < 5 years were more likely to have experienced a previous malaria episode in both the intention-to-treat (OR: 2.83; 95%CI 1.94–4.11; p-value: < 0.001) and per-protocol (OR: 2.69; 95%CI 1.79–4.03; p-value: < 0.001) analyses. Considering age as continuous variable, each one-year increase in age was associated with a 2% decrease in the odds of experiencing previous malaria episodes during the study period (OR: 0.98, 95%CI 0.97–0.99: p-value: < 0.001). The association was also observed considering seasonality. Participants diagnosed with malaria during peak season were more likely to have experienced a previous malaria episode in both the intention-to-treat (OR: 1.41; 95%CI 1.10–1.84; p-value: 0.006) and the per-protocol (OR: 1.31; 95%CI 1.05–1.82; p-value: 0.019) analyses. Although participants with low BMI presented higher odds of having a previous malaria episode (OR: 1.60; 95%CI 1.20–2.12; p-value: 0.001) in the intention-to treat population, this was not observed in the per-protocol analysis.

### Crude association between previous malaria episodes and treatment failure

**Fig. 2:**
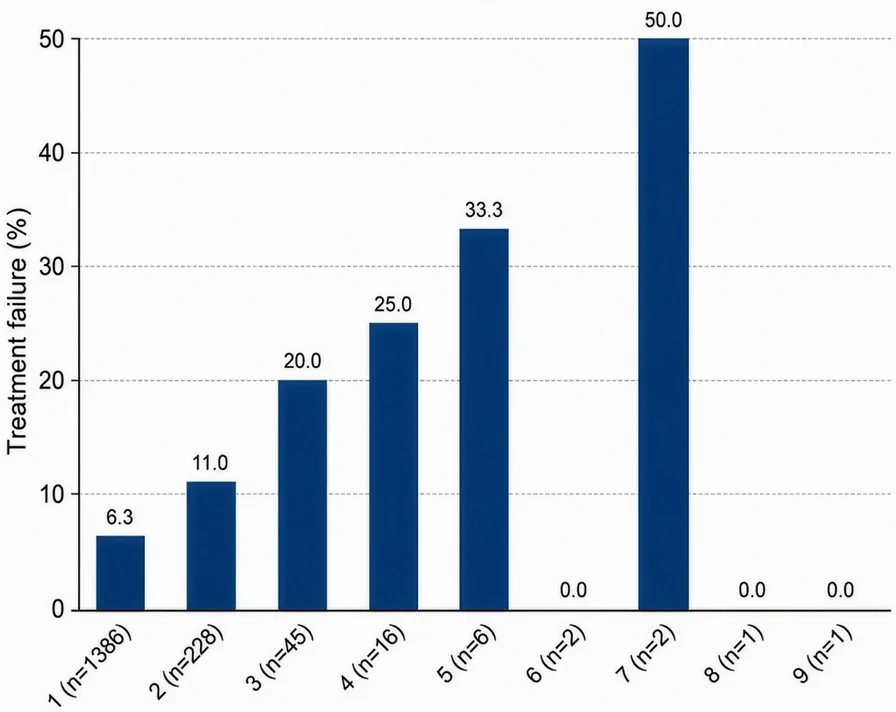
Treatment failure by malaria episode

In the per-protocol analysis, individuals with any prior malaria episode within the study period had 2.31 (95%CI 1.55–3.43; p-value: < 0.001) times the odds of presenting recurrence and 2.78 (95%CI 1.16–6.69; p-value: 0.022) times the odds of recrudescence compared to those with no previous episodes. Additionally, there was evidence of trend, evidenced by a 2.51-fold (95%CI 1.76–3.57; p-value: < 0.001) and 3.60-fold (95%CI 1.57–8.24; p-value: 0.003) increase in the odds of recurrence and recrudescence, respectively, the higher the number of malaria episodes. These findings are described in Table 3. Figure 2 shows the increasing proportion of recurrence according to the number of the episode. Conversely, the association between previous malaria episodes and treatment failure was not observed in the intention-to-treat analysis. The median time interval between the current episode that resulted in treatment failure and the previous episode did not statistically differ comparing participants with and without recurrence (112.5 days; IQR: 65–165.5 vs 96.5 days; IQR: 57–201) or recrudescence (126.5 days; IQR: 97–160 vs 97.5 days; IQR: 57–194) in per-protocol analyses.

**Table 3:** Unadjusted logistic regression results assessing the association between previous malaria episodes and treatment failure

	Intention-to-treat			Per-protocol		
	Recurrence n (%) (N = 375)	OR (95%CI)	p-value	Recurrence n (%) (N = 129)	OR (95%CI)	p-value
Previous malaria						
No	298 (18.3)	–	0.073	89 (6.4)	–	< 0.001
Yes	77 (22.5)	1.30 (0.98–1.72)		40 (13.6)	2.31 (1.55–3.43)	
Per malaria episode						
1 st episode	296 (18.2)	–	0.075	88 (6.4)	–	< 0.001
2nd episode	57 (21.4)	1.22 (0.88–1.67)		25 (11.0)	1.82 (1.14–2.90)	
≥ 3rd episode	22 (27.9)	1.73 (1.04–2.88)		16 (21.9)	4.14 (2.28–7.51)	
Test for trend of odds	–	1.30 (1.04–1.63)	0.021	–	2.51 (1.76–3.57)	< 0.001

	Recrudescence n (%) (N = 277)	OR (95%CI)	p-value	Recrudescence n (%) (N = 22)	OR (95%CI)	p-value
Previous malaria						
No	229 (14.1)		0.985	14 (1.0)		0.022
Yes	48 (14.0)	1.00 (0.71–1.39)		8 (2.8)	2.78 (1.16–6.69)	
Per malaria episode						
1 st episode	228 (14.1)	–	0.996	14 (1.0)	–	0.031
2nd episode	38 (14.2)	1.02 (0.70–1.47)		4 (1.8)	1.77 (0.58–5.41)	
≥ 3rd episode	11 (13.9)	0.99 (0.52–1.90)		4 (5.6)	5.74 (1.84–7.90)	
Test for trend of odds	–	1.00 (0.78–1.29)	0.978	–	3.60 (1.57–8.24)	0.003

Proportions of treatment failure defined as recurrence per malaria episode category in the per protocol population.

### Multivariate logistic regression models

In the multivariate analysis, the association observed in the crude analysis remained after adjustment for age, BMI, and seasonality. Age was modelled as a continuous variable as a significant linear trend with the outcome was observed. Individuals with any prior malaria episode within the study period had 1.96 (95%CI 1.31–2.94; p-value: 0.001) times the odds of presenting recurrence and 2.44 (95%CI 1.01–5.94; p-value: 0.049) times the odds of recrudescence compared to those with no previous episodes in the per-protocol analyses. These findings are shown in Table 4. The association between prior malaria and treatment failure indicated a reduction in the effect with increasing age when modified by age treated as a continuous variable (interaction OR: 0.70, 95%CI 0.49–0.99; p-value: 0.045). No evidence of effect modification was observed when age was modelled as a categorical variable (< 5 vs ≥ 5 years). A mixed-effects model accounting for repeated observations yielded a negligible random intercept variance, indicating minimal within-participant clustering, and results were unchanged. Therefore, the standard logistic model was retained.

**Table 4:** Multivariate logistic regression models

Models	Intention-to-treat		Per-protocol	
	OR (95%CI)	p-value	OR (95%CI)	p-value
Model 1: Recurrence Adjusted for age, BMI, seasonality	1.22 (0.92–1.63)	0.171	1.96 (1.31–2.94)	0.001
Model 2: Recrudescence Adjusted for age, BMI, seasonality	0.98 (0.70–1.37)	0.887	2.44 (1.01–5.94)	0.049

## Discussion

This study conducted in a holoendemic malaria area is the first to our knowledge assessing the association between previous malaria episodes and current malaria treatment failure in a cohort study. Our findings show that having had previous malaria during the study period was associated with 96% increased odds of recurrence and 144% increased odds of recrudescence by day 28 in the adjusted per-protocol analysis. Notably, this association reduced with increased age. Furthermore, an increasing number of previous malaria episodes was associated with progressively higher odds of current treatment failure. In addition, younger age was associated with higher odds of both multiple malaria episodes and treatment failure on day 28. Although the association between previous malaria episodes and treatment failure was not observed in the intention-to-treat analysis, this could be explained by the handling of missing day 28 endpoints. Participants with a missing endpoint were classified as treatment failures, which may have resulted in misclassification and diluted the observed association.

Although there are many publications evaluating predictors of treatment failure, few include prior malaria episodes as a variable. Nevertheless, similar findings have been reported. A prospective study conducted in camps for displaced persons in Thailand assessed treatment outcomes of children and adults with uncomplicated malaria, diagnosed by blood smear and treated with mefloquine. Participants who had received mefloquine 30–61 days before and in the preceding month presented a 1.59-fold increased risk (95%CI 1.31–1.92) of parasitological treatment failure compared to the participants who had not required antimalarial medication in the previous 2 months [11]. Another facility-based prospective study conducted in Ethiopia assessed treatment outcomes in adults with uncomplicated malaria by any *Plasmodium* species diagnosed both clinically and by blood smear and treated according to the National Guidelines. Participants with a previous malaria episode presented a 18.62-fold increased risk (95%CI 5.15–67.25) of unsuccessful treatment outcome compared to those without a previous malaria episode, although such outcome comprised either parasitological or clinical parameters [24]. Conversely, antimalarial use in the preceding 6 weeks was not identified as risk factor for treatment failure in a study in Nigeria. This secondary data analysis from 4 clinical trials conducted at a university hospital in Ibadan assessed predictors of failure of treatment with sulfadoxine-pyrimethamine in children with uncomplicated falciparum malaria [33]. Contrary to the analysis conducted in our study, those three studies relied on self-reporting for the determination of previous malaria episode, prone to recall bias and misclassification.

The association between recurrence and having previous malaria episodes may be partially explained by the fact that people living in locations with particularly high transmission are more prone to early reinfections. In this scenario, a positive thick blood smear on day 28 may in fact reflect a new malaria episode. Therefore, this analysis presented both, microscopic reappearance of parasites as well as PCR-confirmed recrudescence. Although treatment resistance to ACTs is a possibility, it is an unlikely contributing factor in this context considering that it has not been described in Gabon yet. It is noteworthy to highlight that in real life practice recurrence entails a new treatment course irrespective of the cause. Hence, the identification of individuals more prone to this outcome enables a tailored surveillance and intervention. The response to antimalarial therapy involves a complex interaction of several factors. These include parasite characteristics, transmission intensity, pharmacokinetic and pharmacodynamic properties of the drug, and host immunity. An impaired immunological response can be associated with higher risk of treatment failure [34]. This is evidenced by the higher risk of recrudescence among HIV-infected adults with low CD4-T-cell counts [35]. Additionally, young age alone may constitute a risk factor for treatment failure, which may be explained by the ineffective immune system and lack of naturally acquired immunity [36, 37]. Following this logic, more previous malaria episodes may lead to greater immunity and thus a lower risk of treatment failure. However, our findings indicate that, even after PCR correction, which excludes reinfections, treatment failure measured as recrudescence is still associated with the number of previous documented malaria episodes. In our analysis, the higher the number of previous malaria episodes, the higher the odds of treatment failure for the subsequent episode.

The increased odds of recrudescence in participants with previous malaria may be related to transient immunological alterations following *Plasmodium* infection, as previously described [38, 39]. However, this proposed immunological mechanism cannot be directly substantiated by the present study, as no immunological parameters were assessed. *P. falciparum* parasites regulate the host immune response by impairing both humoral and cellular immunity [40–42]. This mechanism is complex, yet thoroughly described among others in the context of vaccination [43]. For example, in a study using a recombinant viral-vectored vaccine, parasitemia before vaccination suppressed the T cell response measured by interferon-γ production [44]. Additionally, there is evidence that the immunological response to vaccination with heterologous polysaccharide antigen is impaired in children with clinical malaria or asymptomatic parasitemia. Conversely, children protected from *P. falciparum* infection by chemoprophylaxis or sickle-cell trait presented increased antibody responses to meningococcal polysaccharides than unprotected children in highly endemic regions [45–48]. The positive result of this inflammatory response downregulation is a reduction of harmful consequences of immune overactivation and a lower risk of disease and death. This may contribute to tolerance to the parasite, as evidenced by asymptomatic parasitemia common among adults from highly endemic areas [43, 49]. In the long term, repeated malaria episodes may contribute to an immunological balance that promotes asymptomatic tolerance to the parasite without negatively affecting subsequent antimalarial treatment response. However, in the short term, transient immunosuppression following a malaria episode could negatively affect the treatment outcome of a subsequent episode. This immunosuppressive effect usually lasts for about 2–4 weeks in uncomplicated and 8 weeks in severe malaria, increasing the vulnerability of the host to reinfection, especially in children [50, 51]. Interestingly, in our study the median time interval between the current episode that resulted in treatment failure and the previous episode was longer than the duration of transient immunosuppression described in the literature (recurrence 16.1 weeks; recrudescence 18.1 weeks). This suggests that transient immunosuppression alone may not fully explain the observed association. Other unmeasured factors may also contribute, as previous malaria episodes may reflect differences in malaria exposure, socioeconomic conditions, healthcare access, or health-related behaviors that were not assessed in the present study.

The main strength of this study is its prospective cohort design objectively documenting the number of previous malaria episodes in the context of the trial. By not relying on self-reported questionnaires, it minimized the risk of recall bias and misclassification. Additionally, a high proportion of children aged < 5 years old were included, increasing the generalizability of the findings to this age group. Finally, the availability of PCR genotyping ensured an accurate definition of treatment failure. However, PCR correction resulted in a substantial reduction in the number of outcome events. This limited the statistical power and precision of the multivariable analysis. Another limitation was that the hypothesis tested was not an a priori hypothesis of the main study. Although medical history was collected using a questionnaire, several potentially relevant confounders were not systematically evaluated. These included HIV status, CD4 count, co-morbidities, and other reasons for immunosuppression, such as autoimmune or advanced chronic diseases.

Although this study reinforces the concept that having previous malaria episode is associated with subsequent treatment failure, its mechanism and clinical implication remain unclear. Moreover, it is unclear what time interval between two malaria episodes is considered critical. To elucidate these questions, prospective studies with a larger sample size and longer follow-up are recommended. Such studies should document every new episode and assess different treatment regimens in real-world settings, including vulnerable groups such as immunosuppressed and pregnant women. Immunological analysis could provide further insight into how the immunological alterations are correlated with clinical aspects. These findings could potentially inform chemoprevention strategies for this subset of patients.

## Conclusions

In conclusion, having a documented previous malaria episode within the study period was associated with subsequent antimalarial treatment failure. This effect was more evident at younger age and increased proportionally to the number of episodes. Transient immune alterations following Plasmodium infection may contribute to this association, although other unmeasured factors may also play a role. This understanding may improve clinical malaria management in highly endemic regions, especially during peak season, by identifying those patients at highest risk for treatment failure.

## Data Availability

The data underlying the results presented in the study are available from Medicines for Malaria Venture (mmv.org) on reasonable request.

## Abbreviations

ACT: Artemisinin-based combination therapy
BMI: Body mass index
CHW: Community health worker
CI: Confidence interval
HIV: Human immunodeficiency virus
IQR: Interquartile range
OR: Odds ratio
*P.*: *Plasmodium*
PCR: Polymerase chain reaction
WHO: World Health Organization
